# Status Quo Bias: An Overlooked Barrier to Sustainable Surgical Care

**DOI:** 10.1111/ans.70887

**Published:** 2026-07-27

**Authors:** Chen Lew, Arun Dhir, Ben Dunne, Eugenie Kayak, Robert Hoffmann

**Affiliations:** ^1^ Department of General Surgery, Department of Health and Human Services Royal Hobart Hospital Hobart Tasmania Australia; ^2^ Epworth HealthCare Melbourne Victoria Australia; ^3^ Parkville Campus University of Melbourne Parkville Victoria Australia; ^4^ Department of Critical Care The University of Melbourne Parkville Australia; ^5^ Tasmanian Behavioural Lab University of Tasmania Hobart Tasmania Australia

**Keywords:** bias, surgery, sustainability

## Abstract

Healthcare contributes to the climate change, with operating theatres among the most resource‐intensive environments. Although technological solutions to mitigate this exist, behavioural factors remain under‐recognised barriers to sustainable surgical practice. This perspective examines status quo bias as a driver of reliance on single‐use devices in surgery, one of the contributors to the carbon footprint of operating rooms. Drawing on behavioural economics, historical examples, and surgical data, we explore how entrenched defaults, training culture, and lack of feedback perpetuate waste without improving outcomes. Addressing status quo bias through redesigned defaults, feedback mechanisms, and cultural change is essential to advancing responsible surgical care.

## 1 Introduction

Healthcare is a paradoxical industry: A sector devoted to protecting life that is also a major contributor to the climate crisis and thus contributes to adverse health outcomes. Operating theatres are at the heart of this paradox. They consume three to six times more resources than other hospital departments and they generate a significant volume of waste in proportion to the wider hospital system—predominantly from energy, consumables and single‐use devices (SUDs) [1]. For this reason, operating theatres present several opportunities for waste and emissions reduction in healthcare [2].

We propose that the barriers to sustainable surgical care are not just technological or logistical, but also psychological. A key, under‐recognised barrier is status quo bias (SQB): The tendency to persist with established routines even when alternatives may be more effective or sustainable. Recognising and overcoming SQB may therefore be necessary for the evolution of surgical care into a climate‐responsible discipline.

### 1.1 Understanding Status Quo Bias

SQB was introduced and demonstrated experimentally by Samuelson and Zeckhauser, who showed that individuals disproportionately favour existing states or default options even when alternatives may be superior [3]. They attributed this tendency to switching costs, uncertainty, loss aversion, anticipated regret, and commitment to prior decisions [3]. While these mechanisms can make maintaining the status quo attractive, they may also discourage beneficial change.

In surgery, SQB may manifest as adherence to established operative preferences, including the use of SUDs. Despite often lacking outcome superiority, SUDs may be favoured for perceived advantages in infection control, reliability, and convenience over reusable alternatives. The reliance on SUDs may therefore reflect inertia rather than necessity, warranting closer scrutiny of the role SQB plays in shaping such practices [4].

### 1.2 Status Quo Bias in Healthcare Practice

Experimental studies suggest clinicians often favour existing treatment approaches or inaction, even when alternatives may offer superior outcomes [5, 6]. Historical examples include the prolonged resistance to hand hygiene following Semmelweis' observations [7]. Similar patterns are evident in the implementation of Enhanced Recovery After Surgery (ERAS) protocols, where uptake has remained slow despite a substantial evidence base demonstrating improved patient outcomes, with resistance to change recognised as one of several barriers to implementation [8]. Together, these findings suggest SQB may slow the adoption of evidence‐based practices and innovations.

Consistent with this concept, observational work has demonstrated substantial variation in discretionary single‐use device utilisation between surgeons performing the same procedure, without corresponding differences in clinical outcomes [9]. These findings suggest that resource use is often driven by habit and inherited practice rather than clinical necessity [3, 6, 9, 10].

Yet such variation often goes unrecognised, with hospitals rarely providing feedback on device consumption or associated costs, leaving clinicians unaware of peer performance. Although capacity to analyse these data is expanding through electronic device tracking, integration with electronic medical records, and AI‐enabled analytics, such tools are rarely used to provide meaningful feedback on the environmental and economic footprint of surgical practice [11, 12].

Our specialist training systems may further reinforce status quo bias [13, 14]. Surgical training (unintentionally) follows an apprenticeship model in which trainees adopt the practices and preferences of senior surgeons they train with, often through implicit rather than explicit instructions [13, 14]. Over time, these inherited defaults become embedded within institutional culture and resistant to scrutiny [13, 14]. The result can be a cycle where resource‐intense devices, such as many SUDs may be preferred over more environmentally friendly choices, with little opportunity to reflect on the real outcomes they may deliver.

### 1.3 Why Does Status Quo Bias Persist?

SQB is common in healthcare and other decision domains because it is a consequence of how humans tend to make decisions. To deal with complex environments, people resort to fast, automatic decision heuristics, including relying on familiar patterns and past experience. This type of cognition is known as fast thinking, or System 1 [10]. Although these heuristics can sometimes produce systematically inefficient decisions, they are generally ecologically rational, meaning they are well adapted to the real‐world environments and constraints within which such decisions are made [15].

Several well‐described cognitive mechanisms contribute to the persistence of SQB. Loss aversion causes clinicians to perceive the risks of changing established practice as greater than the potential benefits. Switching costs, including the time and effort required to learn new techniques or workflows, further discourage change. Long‐standing professional investment creates attachment to familiar practices, while habitual routines reduce cognitive effort in busy clinical environments. Together, these mechanisms favour continuation of existing practice, particularly in surgery where decisions are often made under time pressure and uncertainty.

Surgery combines many of the conditions that encourage reliance on fast thinking, including time pressure, information overload, uncertainty, and high stakes [16]. In these circumstances, familiar techniques and established ways of working can feel safer and easier to justify than change, even when evidence supports an alternative approach.

Importantly, reliance on existing practice is not always irrational. As originally noted by Samuelson and Zeckhauser, remaining with the current approach may sometimes be a reasonable response to uncertainty, limited information, or the practical costs of change [3]. The challenge is to distinguish between situations where the status quo remains justified and those where it prevents the adoption of better practice.

### 1.4 Overcoming Status Quo Bias

Overcoming SQB requires coordinated organisational and behavioural interventions that extend beyond awareness‐raising alone [17]. Several implementation and behavioural frameworks could be applied to sustainable surgery, including COM‐B, the Behaviour Change Wheel, and the Theoretical Domains Framework [17, 18, 19, 20]. We highlight the EAST framework, Easy, Attractive, Social, and Timely, because it is deliberately pragmatic, simple to operationalise, and has been widely adopted in health policy to facilitate behavioural change through modifications to defaults, social norms and decision environments rather than relying solely on education [17, 19, 20]. Potential strategies to improve surgical sustainability within the EAST framework include:

#### 1.4.1 Easy

Sustainable choices should become the default wherever possible. Procurement policies that prioritise reusable alternatives and redesigned surgeon preference cards can reduce unnecessary reliance on discretionary single‐use devices [11, 12].

#### 1.4.2 Attractive

Providing clinicians with feedback on the environmental and financial consequences of device utilisation, supported by AI‐enabled monitoring and benchmarking, may increase engagement with sustainable practice [11, 12].

#### 1.4.3 Social

Sustainability is more likely to become embedded when respected surgeons model environmentally responsible practice, institutions explicitly value sustainability alongside patient safety, and performance is benchmarked transparently across teams.

#### 1.4.4 Timely

Behavioural interventions should target periods when practice is naturally changing, including procurement reviews, theatre redesign, and surgical training, when clinicians may be more receptive to adopting new habits.

These interventions collectively target the roots of SQB and provide opportunities for implementation on an individual or institutional level. By changing defaults, exposing hidden variation, and aligning values, we believe these changes have the potential to shift the surgical culture towards greater sustainability.

## 2 Conclusion

Status quo bias is a silent but powerful barrier to environmentally sustainable surgical care. By perpetuating reliance on familiar patterns of resource consumption and practices perceived to be safer despite emerging evidence supporting alternative approaches, SQB may hinder the adoption of more sustainable alternatives. Addressing SQB requires coordinated system‐level action and a re‐envisioning of how sustainable surgical care is designed and delivered. History has shown that resistance to change, from the adoption of hand hygiene to ERAS protocols, has delayed progress with significant consequences. Today, the stakes are planetary.

## Author Contributions

C.L. contributed to conceptualisation, literature review, writing original draft, and review and editing. A.D. and R.H. contributed to conceptualisation, supervision, and review and editing. B.D. and E.K. contributed to review and editing. All authors reviewed and approved the final manuscript.

## Funding

The authors have nothing to report.

## Conflicts of Interest

The authors declare no conflicts of interest.

## Data Availability

Data sharing not applicable to this article as no datasets were generated or analysed during the current study.
